# Ebola Virus Stability on Surfaces and in Fluids in Simulated Outbreak Environments

**DOI:** 10.3201/eid2107.150253

**Published:** 2015-07

**Authors:** Robert Fischer, Seth Judson, Kerri Miazgowicz, Trenton Bushmaker, Joseph Prescott, Vincent J. Munster

**Affiliations:** National Institutes of Health, Hamilton, Montana, USA

**Keywords:** Ebola virus, viruses, environment, stability, surfaces, fluids, simulated outbreak environments

## Abstract

We evaluated the stability of Ebola virus on surfaces and in fluids under simulated environmental conditions for the climate of West Africa and for climate-controlled hospitals. This virus remains viable for a longer duration on surfaces in hospital conditions than in African conditions and in liquid than in dried blood.

Since March 2014, >22,000 cases of Ebola virus disease (EVD) and ≈ 9,000 deaths have been reported in West Africa (*1*). Thousands of health care professionals have been mobilized to West Africa to assist with the ongoing outbreak of EVD (*2*). More than 800 Ebola virus (EBOV) infections have been reported in health care professionals (*1*).

Determining the persistence of EBOV on surfaces and under environmental conditions specific to outbreak settings and disease-endemic areas is critical to improving safety practices for these health care workers (*3*), as well as answering questions about EBOV transmission among the public (*4*). Researchers have experimentally assessed the stability of other EBOV strains on plastic, glass, and steel within dried media or guinea pig serum (*5*); in the dark on glass (*6*); and during exposure to UV light (*7*). However, the environmental conditions of these studies do not reflect the higher temperatures and relative humidities (RHs) in outbreak regions, or the current outbreak strain. No infectious EBOV could be found during environmental sampling in a ward with EVD patients; however, this result could be more indicative of cleaning measures than actual virus stability (*8*).

We report stability of EBOV with a current outbreak strain from Guinea (Makona-WPGC07) (*9*) on 3 clinically relevant surfaces: stainless steel, plastic, and Tyvek (Dupont, Wilmington, DE, USA). We also determined the stability of EBOV in water, spiked human blood, and blood from infected nonhuman primates (NHPs). These experiments were conducted in 2 environmental conditions, 21°C, 40% RH, and 27°C, 80% RH, to simulate a climate-controlled hospital and the environment in West Africa, respectively.

## The Study

We tested the stability of EBOV on 3 materials commonly found in an Ebola treatment unit (ETU) in West Africa: 1) utility-grade (308) stainless steel washers (McMaster-Carr, Atlanta, GA, USA); 2) plastic (Teflon [polytetrafluoroethylene]; McMaster-Carr); and 3) Tyvek (from the front of a coverall). For each time point, 3 disks (4-cm diameter) of each material were placed individually into wells of a 6-well plate. Five samples (10 μL/sample) containing a total dose of 10^6^ 50% tissue culture infectious doses (TCID_50_s) of EBOV in cell-free medium were evenly distributed on the disks. The plates were divided into groups, and each group was placed into a plastic HEPA-filtered box and placed at 21°C, 40% RH, or 27°C, 80% RH. The samples were dried naturally, and virus titers were determined over a 14-day period.

In the surface and fluid stability experiments, all samples were stored at −80°C until titration (1 freeze–thaw cycle of EBOV samples that did not change virus titer). Titrations were performed on Vero E6 cells as described (*10*,*11*). The TCID_50_ per milliliter for each sample at each time point was calculated by using the Spearman-Karber method (*11*).

**Figure 1:**
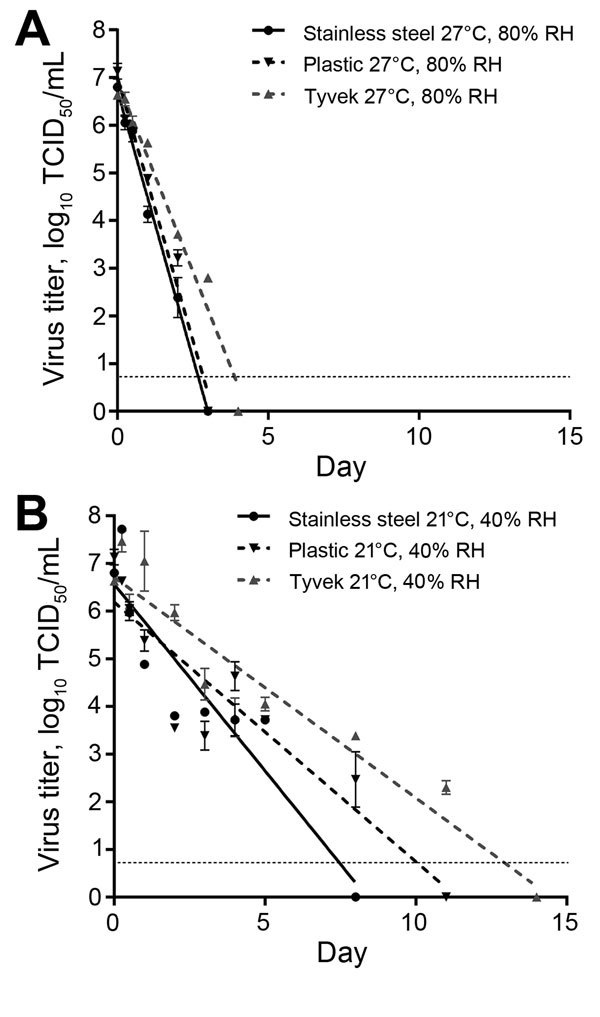
Linear regression model showing the effect of different environmental conditions and surfaces on survival of Ebola virus (EBOV). Virus was dried on 3 surfaces found in outbreak settings at A) 27°C, 80% relative humidity (RH) (West African tropical conditions) and B) 21°C, 40% RH (climate-controlled hospital conditions). Virus concentration was reduced at a significantly slower rate on all surfaces in hospital conditions than in tropical conditions (p<0.0001 for all surfaces). Triplicate samples were taken at each time point. Error bars indicate mean ± SEM virus titer. Dashed line indicates the limit of detection for the assay. An analysis of covariance equivalent test was used to compare linear regression models and determine differences in virus reduction rates. TCID_50_, 50% tissue culture infectious dose.

Because viral decay rates often exhibit first-order kinetics (*12*), we log_10_ transformed our TCID_50_ calculations to represent virus titer and used a linear regression analysis (Prism version 6.05; GraphPad, San Diego, CA, USA) to determine the log_10_ reduction rate of EBOV on each surface at both environmental conditions (Figure 1; Table 1). We also determined whether linear regression models were significantly different from each other at the p<0.05 level by using an analysis of covariance equivalent test in Prism. Overall, virus remained viable longest in hospital conditions and on Tyvek. Viable EBOV was detectable for 3 days on Tyvek at tropical conditions.

**Table 1:** Linear regression models for survival of Ebola virus on surfaces and in fluids at different environmental conditions*

Condition	Temperature, °C	Relative humidity, %	Model†	r^2^	Virus log reduction time, d‡
Stainless steel	27	80	Y = −2.240X + 6.729	0.9798	0.45
Stainless steel	21	40	Y = −0.7829X + 6.564	0.8544	1.3
Plastic	27	80	Y = −2.205X + 7.008	0.9745	0.45
Plastic	21	40	Y = −0.5445X + 6.188	0.8303	1.8
Tyvek	27	80	Y = −1.599X + 6.939	0.9713	0.63
Tyvek	21	40	Y = −0.4631X + 6.709	0.8878	2.2
Drying human blood	27	80	Y = −0.6806X + 4.951	0.8724	1.5
Drying human blood	21	40	Y = −0.6917X + 4.828	0.9037	1.5
Liquid human blood	27	NA	Y = −0.1148X + 4.651	0.2892	8.7
Liquid human blood	21	NA	Y = −0.05000X + 4.231	0.05293	20
Water	27	NA	Y = −1.133X + 4.483	0.9607	0.88
Water	21	NA	Y = −0.5694X + 4.201	0.9139	1.8

*NA, not applicable. †Y, log_10_ 50% tissue culture infectious dose/mL; X, days.  ‡In hospital conditions, virus titer on steel was reduced significantly faster than on plastic (p = 0.004) and on Tyvek (p<0.0001), but there was no significant difference in reduction between Tyvek and plastic (p = 0.13). In tropical conditions, there was no significant difference in virus titer reduction on steel and on plastic (p = 0.78). However, virus decayed more slowly on Tyvek than on steel (p<0.0001) and on plastic (p<0.0001). There was no significant difference in reduction rate in virus titer in drying human blood in hospital or tropical conditions (p = 0.92). Stability of virus in liquid blood did not fit a linear regression model. Virus was reduced significantly faster at 27°C than in water at 21°C (p = 0.0001).

**Figure 2:**
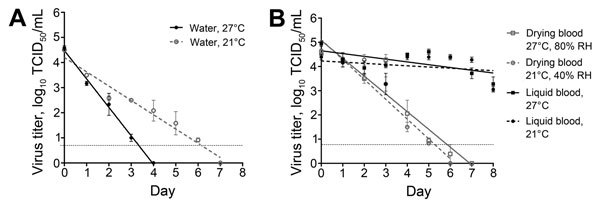
Linear regression model showing stability of Ebola virus (EBOV) in fluids under different environmental conditions. A) EBOV stability in water at 2 environmental temperatures. Virus concentration was reduced at a significantly faster rate in 27°C water than in 21°C water (p = 0.0001). B) Stability in drying or liquid EBOV-spiked human blood samples at 2 environmental conditions. Virus concentration was reduced at a significantly faster rate by drying than in liquid blood at both conditions (p<0.0001 for each condition). No significant difference between reduction rates in virus titer in drying human blood at both conditions was found (p = 0.92). Triplicate samples were taken at each time point. Error bars indicate mean ± SEM virus titer. Dashed line indicates the limit of detection for the assay. An analysis of covariance equivalent test was used to compare linear regression models and determine differences in virus reduction rates. TCID_50_, 50% tissue culture infectious dose.

The stability of EBOV in water was assessed by diluting 150 µL virus stock in 2.85 mL of Ambion diethylpyrocarbonate–treated water (Thermo Fisher Scientific, Pittsburgh, PA, USA) and removing residual protein and medium with 1 initial and 2 rinse spins on Amicon Ultra Centrifugal Filters 100K MWCO (Merck, Darmstadt, Germany). EBOV was more stable in water at 21°C and had an ≈1 log_10_ reduction/day in water at 27°C (Table 1; Figure 2, panel A).

The stability of EBOV in human blood was assessed by spiking blood samples from a healthy human volunteer to achieve a 10^5^ TCID_50_/mL virus titer. The spiked blood was distributed in 1-mL aliquots into closed screw-top vials to maintain a liquid state, spread in 50-µL aliquots onto the bottom of a 24-well plate, and dried. One group of samples was stored at 21°C, 40% RH, and the other group was stored at 27°C, 80% RH. EBOV stability in drying blood exhibited first-order kinetics and was viable for up to 6 days at tropical conditions (Table 1; Figure 2, panel B).

To approximate the stability of EBOV in naturally infected human blood, we used blood from cynomolgus macaques (*Macaca fascicularis*) as a proxy. Blood was collected during necropsy from 3 macaques that were previously enrolled in an Animal Care and Use Committee–approved EBOV pathogenesis study and were euthanized because they exhibited signs of EVD and viremia. Blood samples were divided into 2 groups with 2 sets of 150-μL aliquots for each time point; each group was stored at the conditions described above with each set in the liquid or drying state, and virus viability was assessed over a 14-day period. Because of variation in calculated virus titer from each of the individual NHPs, the log_10_ reduction rate could not be approximated and only the initial titer and the duration of viability are shown (Table 2). In general, EBOV maintained viability for a longer duration in liquid than in drying blood regardless of initial titer or environmental condition.

**Table 2:** Stability of Ebola virus in infected nonhuman primate blood under different environmental conditions*

Blood sample, condition	Initial virus titer, log_10_ TCID_50_/ mL	No. days viable
NHP 1		
Drying 27°C, RH 80%	6.5	5
Drying 21°C, RH 40%	6.5	1
Liquid 27°C	7.2	14
Liquid 21°C	7.2	14
NHP 2		
Drying 27°C, RH 80%	2.8	5
Drying 21°C, RH 40%	2.8	1
Liquid 27°C	4.2	11
Liquid 21°C	4.2	1
NHP 3		
Drying 27°C, RH 80%	7.2	4
Drying 21°C, RH 40%	7.2	4
Liquid 27°C	6.5	8
Liquid 21°C	6.5	14


*TCID_50_, 50% tissue culture infectious dose; NHP, nonhuman primate; RH, relative humidity.

## Conclusions

We found that EBOV can persist on surfaces common in an ETU, highlighting the need for adherence to thorough disinfection and doffing protocols when exiting the ETUs and careful handling of medical waste. In addition, EBOV maintains viability for a longer duration in liquid than in dried blood. EBOV in blood of experimentally infected NHPs persists for a similar duration as EBOV in spiked human blood. A recent study showed that blood in the body cavity of an NHP contained viable EBOV for up to 7 days after death (*13*). We detected viable EBOV in drying blood for up to 5 days at both environmental conditions in human and NHP blood. Therefore, dried and liquid blood from an infected person in their home or ETU should be treated as potentially infectious. The finding that EBOV remains viable in water for as long as 3 (27°C) or 6 (21°C) days at the experimental concentration warrants further investigation into the persistence of the virus in aqueous environments, such as in wastewater or sewage canals. Viable EBOV has been isolated from urine (*14*) but not from human stool (*8*). Therefore, the potential for dissemination of EBOV through wastewater remains unknown.

This study is subject to several limitations. First, because standard volumes for samples were used, different volumes or matrices could influence the stability of EBOV under the tested conditions. Second, blood samples from the NHPs might have different immunologic or biochemical conditions, which can potentially influence virus stability. Third, the experimental conditions in the laboratory are sterile, but in disease-endemic areas and ETUs, bacteria or chemicals could influence EBOV viability.

Overall, we found that different environmental conditions, fluids, and surfaces influence the persistence of EBOV. These findings demonstrate that such factors are crucial in understanding transmission and improving safety practices.
